# Structure–Activity Relationship of Ionic Liquids for Acid Corrosion Inhibition

**DOI:** 10.3390/ijms26125750

**Published:** 2025-06-16

**Authors:** Aymane Omari Alaoui, Mouslim Messali, Walid Elfalleh, Belkheir Hammouti, Abderrahim Titi, Fadoua El-Hajjaji

**Affiliations:** 1 Systems Engineering, Modeling and Analysis Laboratory (LIMAS), Faculty of Sciences Dhar El Mahraz, Sidi Mohamed Ben Abdellah University, BP 1796 Atlas, Fez 30000, Morocco; omariayman18@gmail.com; 2 Department of Chemistry, College of Science, Imam Mohammad Ibn Saud Islamic University (IMSIU), P.O. Box 90950, Riyadh 11623, Saudi Arabia; mhmessali@imamu.edu.sa; 3 Department of Biology, College of Science, Imam Mohammad Ibn Saud Islamic University (IMSIU), Riyadh 11623, Saudi Arabia; wbelfallah@imamu.edu.sa; 4 Laboratory of Electrochemistry and Corrosion, Engineering Polytechnic School, Euromed University of Fes (UEMF), Fes 30030, Morocco; hammoutib@gmail.com; 5 Engineering Laboratory of Organometallic, Molecular Materials and Environment (LIMOME), Faculty of Sciences Dhar El Mahraz, Sidi Mohamed Ben Abdellah University, BP 1796 Atlas, Fez 30000, Morocco

**Keywords:** ionic liquids, corrosion inhibitor, electrochemical tests, SEM-EDX analysis, mild steel, DFT, MC

## Abstract

Novel derivatives of imidazolium-based ionic liquids with varying alkyl chains, IL-1, IL-2, and IL-3, were evaluated as corrosion inhibitors for mild steel in 1 M HCl solution. The experimental investigations used Electrochemical Impedance Spectroscopy (EIS) and potentiodynamic polarization (PDP) techniques. The results demonstrated exceptional corrosion inhibition efficiency (>90%), as classified by electrochemical analyses, which identified these corrosion inhibitor compounds as mixed-type. The ionic liquids’ adsorption complied with the Langmuir adsorption isotherm. The characterization of the surface via SEM and EDX confirmed the development of a protective adsorbed inhibitor layer on the steel substrate. Furthermore, the theoretical DFT method (at B3LYP/6-311G (d, p)) was conducted to describe the electronic properties and reactivity of the molecules. The Monte Carlo simulation on the surface of Fe(1 1 0) was assessed to provide in-depth understanding of the adsorption mechanisms and interactions responsible for the corrosion inhibition between the molecules and the surface of the mild steel.

## 1. Introduction

Mild steel (MS) has been extensively used as the main material for pipework in various industrial fields. It is commonly used in applications such as pipes, flow lines, and transport or distribution pipelines in the oil and gas industries. The corrosion of metals is an important economic and safety challenge for various industries, specifically in the fields of construction and oil refining. Acidification is a crucial technique in these industries to promote well formation and improve oil production. However, this process endangers the durability of steel equipment due to acid-induced corrosion [1,2,3,4,5]. The use of corrosion inhibitors is a common practice to protect reactive metal surfaces from degradation in various media. This method remains one of the easiest and most cost-effective methods to reduce the corrosion rate. Organic compounds rich in sulfur, oxygen, and nitrogen, especially those with π-electronic systems, are frequently recognized as effective inhibitors. Numerous studies have shown their ability to provide remarkable inhibitory efficacy [2,5,6,7,8,9,10]. Generally, the inhibitory molecules attach to the metal surface through a chemical or physical adsorption mechanism, which facilitates the creation of a protective film [11,12]. Due to stringent environmental regulations, the adoption of green corrosion inhibitors is strongly encouraged. The development of safe and eco-friendly corrosion inhibitors is actively pursued. Consequently, ionic liquids (ILs) have attracted significant interest from academic researchers and engineers for their crucial, eco-friendly, and cost-effective nature. The physical and chemical properties of ionic liquids are the primary factors in their applications across various fields, including corrosion inhibition. Recently, attention has been directed toward imidazolium ILs, a diverse class of heterocyclic compounds containing nitrogen in their structures, renowned for their ability to form cationic molten salts. The biodegradability, non-toxicity, cost-effectiveness, safety, and high solubility in water render imidazolium-based ILs an ecological and sustainable alternative for numerous industrial applications [13,14,15,16,17].

Quantum chemistry techniques have proven to be very effective in the determination of molecular structures and the analysis of electronic properties. This method allowed for the determination of essential electronic parameters of the molecules. The relationship between the inhibitory effect and the molecular structure of the studied compounds was confirmed. The mechanisms of inhibition specific to organic inhibitors can be elucidated accurately through theoretical modeling approaches. Molecular dynamics (MD) and Monte Carlo (MC) simulations can provide insights into the design of inhibitory systems with optimal properties and elucidate the adsorption mechanism [12,18,19]. This study is focused on investigating the corrosion inhibition efficiency and properties of three imidazolium-based ionic liquids, namely, 3-(2-chlorobenzoyl)-1-phenethyl-1H-imidazol-3-ium chloride(IL-1), 3-(4-chlorobenzoyl)-1-phenethyl-1H-imidazol-3-ium chloride(IL-2), and 3-(4-fluorobenzyl)-1-phenethyl-1H-imidazol-3-ium chloride(IL-3), using electrochemical tests on mild steel in hydrochloric acid medium, as well as surface analysis and quantum chemical calculations DFT and MC.

## 2. Results

### 2.1. Electrochemical Impedance Spectroscopy (EIS)

**Figure 1:**
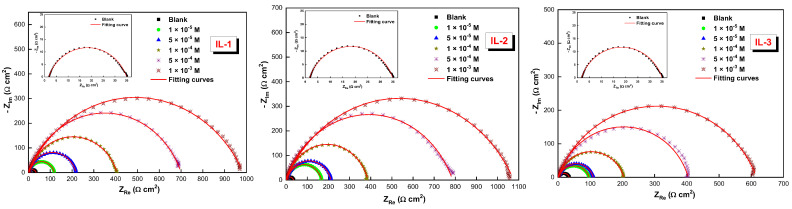
Nyquist diagrams for MS in a 1 M HCl solution at 298 K that has different concentrations of IL-1, 2, and 3.

**Figure 2:**
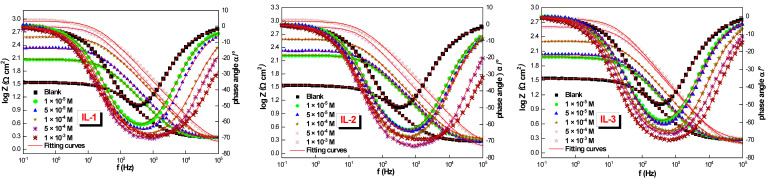
Bode plots for MS in a 1 M HCl solution at 298 K that has different concentrations of IL-1, IL-2, and IL-3.

In the absence of the three inhibitors studied by EIS, the corrosion behavior of mild steel in 1 M of HCl solution was observed. Figure 1 shows the Nyquist plots of the mild steel without and with the addition of the inhibitors at different concentrations of the new ionic liquids studied at 298 K. The Bode plots of the mild steel are also shown in Figure 2. In the medium frequency range, a single-phase peak is detected in the Bode plots [20].

**Figure 3:**
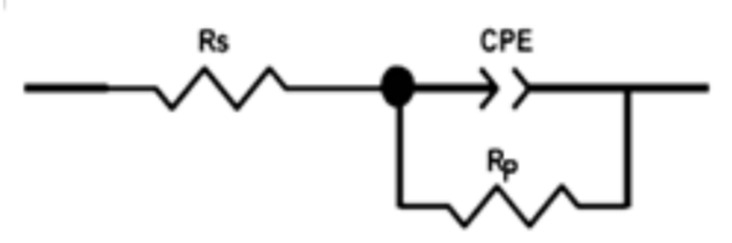
The experimental impedance equivalent circuit.

The resulting graphic representation of the Nyquist graph shows a depressed capacitive half-loop, indicating charge-transfer-controlled corrosion. The capacitive loop has increased due to the roughness and disorganization of the active sites on the mild steel surface [21,22]. To model the EIS results, a circuit similar to Randles’ was utilized. As depicted in Figure 3, the equation was used to calculate the CPE value. The circuit used to fit the experimental data includes the constant phase element (CPE), charge transfer resistance (*R_ct_*), and solution resistance (*R_s_*).

*Z_CPE_* = [*Q(jω)^n^*]^−1^ (1)

*Q* represents the CPE magnitude, ω represents the angular frequency, and i^2^ = −1 is a non-existent number.

Table 1 shows the corrosion kinetic parameters, *C_dl_*, *R_ct_*, and *Q*. The load transfer resistance values increase with the rising concentration of the synthesized products. In contrast, the *C_dl_* values decrease as the concentration increases, which promotes the formation of adsorbed films on the mild steel [23,24] and the replacement of water at the mild steel/solution interface. This leads to a decrease in the thickness of the double electrical layer, in addition to a reduction in the dielectric constant [25,26]. The inhibition efficiency follows the order IL-2 (96.9) > IL-1 (96.6) > IL-3 (94.6) at 10^−3^ M. IL-2 is the most effective inhibitor due to its higher adsorption capacity.

### 2.2. Adsorption Isotherm Study

The efficiency and adsorption capacity of the molecule determine the degree of adsorption of the corrosion inhibitor on the metal surface. Understanding the adsorption mechanism between the studied molecules and the metal is of paramount importance when considering the adsorption process. The adsorption behavior of the imidazolium-derived ILs can be explained by the Langmuir adsorption isotherm and can be described as follows [20]:

(2) $$KC_{inh}=\frac{\theta }{1-\theta }*\frac{C_{inh}}{\theta }\;vs\;C_{inh}$$

**Figure 4:**
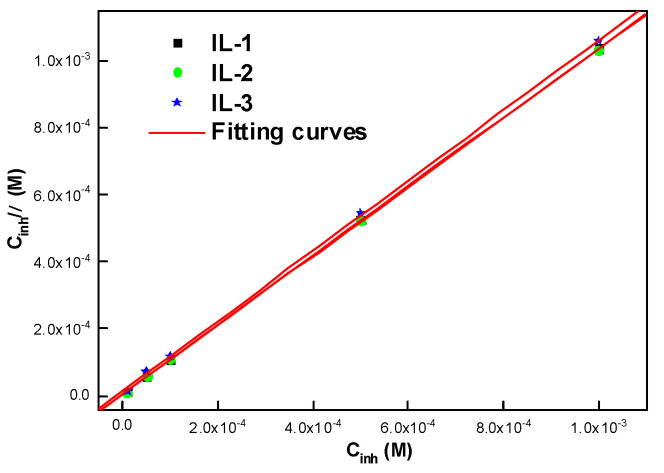
Langmuir adsorption isotherm plots for the mild steel of the ionic liquids (ILs) at 298 K.

Surface coverage is represented by *θ*, *C_inh_* is the concentration of the synthesized ILs (M), and *K* is the adsorption equilibrium constant (L mol^−1^). The plots in Figure 4 show the relationship between *C_inh_* and *C_inh_*/*θ*. In addition, the equation used to calculate Δ*G_ads_* was based on the expression of the standard Gibbs’ free energy of adsorption [27], expressed as

ΔG_ads_ = RTLn (55.5 K) (3)

The solution contains 55.5 moles of H_2_O, and *R* is 8.314 J mol^−1^ K^−1^, while T is the absolute temperature.

Table 2 clearly shows the values of (*R*^2^), the linear correlation coefficients, and the slopes, confirming the Langmuir adsorption model. Thus, these results suggest that the best linear fit for the EIS data is the Langmuir model.

According to studies in the literature, adsorption between the charged inhibitor and the metal is detected when the Δ*G_ads_* value is under or equal to −20 KJ mol^−1^. When the ΔG_ads_ value is equal to −40 KJ mol^−1^ or more, chemisorption may be present. The ΔG_ads_ values are close to −40 KJ mol^−1^ in our case [27,28]. The results obtained demonstrate that the adsorption of the ILs examined is principally chemisorption. They are adsorbed and form strong bonds on the surface of the steel.

### 2.3. Effect of Immersion Time on the ILs Inhibition Efficiency

**Figure 5:**
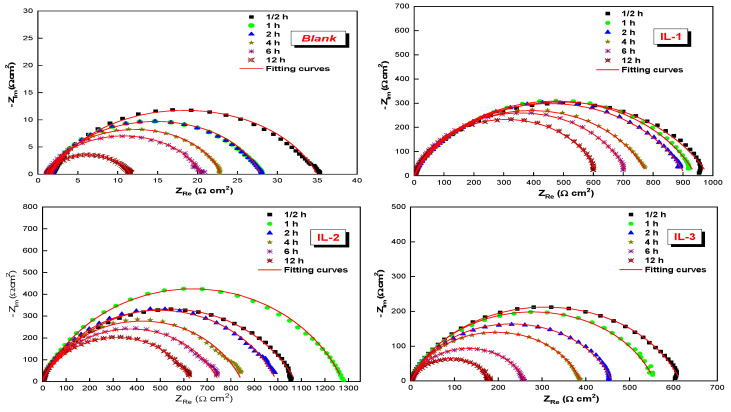
Nyquist diagrams for MS in 1 M HCl with and without the studied ILs after different immersion times.

Several immersion times were used to examine the development in inhibition efficiency of all the ionic liquid compounds examined on mild steel surface corrosion in 1 M of HCl at 298 K. Figure 5 presents the Nyquist curves for MS in a 1 M solution of hydrochloric acid (HCl) without (white) and with 10^−3^ M of ILs during various immersion phases lasting up to 12 h. In addition, Table 3 summarizes the EIS parameters from the Nyquist diagrams. When not all the compounds examined are present, the diameter of the semicircle reduces over time. This reduction in charge transfer resistance is associated with a rise in the double-layer capacitance, which could be due to the accumulation of free metal ions in the diffuse double layer. However, with the existence of ILs, the R_ct_ values decrease over time, while the C_dl_ values increase. We can explain this by the weak permeability of the inhibitor film based on the ionic liquids on the surface of the MS samples. In addition, Table 3 demonstrates that the inhibition efficiency values remained almost unchanged. The value of inhibition efficiency in a half hour of immersion time (Table 3) is smaller or a slightly lower than the value after immersion, confirming the stability of the adsorbed inhibitor film formed by the ionic liquids in an acidic solution of HCl with passing time [20].

### 2.4. Potentiodynamic Polarization (PDP) Measurements

The values of the parameters current density (*i_corr_*), corrosion potential (*E_corr_*), and the anode and cathode (β_a_ and β_c_) Tafel slopes were determined from the anodic and cathodic regions of the Tafel curves. To calculate the corrosion current densities (*i_corr_*), the linear segments of the anode and cathode curves were extrapolated to the corrosion potential. The inhibition efficiency was assessed based on the measured values of icorr using the following relationship.

$${EI}_{PDP}=\frac{i_{corr}^{0}-i_{corr}}{i_{corr}^{0}}$$

where $i_{corr}^{0}$ and *i_corr_* are the corrosion current density in the absence of ILs and the presence of ILs.

**Figure 6:**
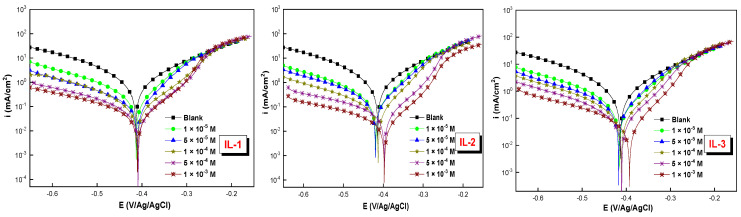
Tafel plots for MS in 1 M HCl solution at 298 K, in the presence and absence of various concentrations of synthesized ILs.

Figure 6 shows the potentiodynamic polarization curves of the metal (MS) in 1 M of HCl, without and with the inhibitors, the ionic liquids, studied at various concentrations. The results in Figure 6 and Table 4 show that the introduction of the imidazolium-based ionic liquid inhibitors into the aggressive acidic medium leads to a simultaneous reduction in the anodic and cathodic current density, with a more pronounced effect as the concentration of inhibitors increases. This suggests that these ionic liquids influence the anodic dissolution of MS steel as well as the cathodic reduction hydrogen evolution mechanism [29,30], while not significantly altering the shape of the Tafel curves. The observed potential shift is attributed to the existence of a corrosion film formed on the surface of the sample. The linear segments of the Tafel curves in the cathodic and anodic regions were extrapolated to the corrosion potential (*E_corr_*) to determine the corresponding corrosion current densities.

According to Table 4, when the compounds are added, the i_corr_ values decrease. This means that increasing the concentration of ILs decreases the i_corr_ values. Also, the results indicate that the maximal values of *η*% for IL-1, IL-2, and IL-3 are 96.8%, 96.6%, and 95.5%, respectively, at 10^−3^ M. The values of *η*% demonstrate that IL-1 is the most effective corrosion inhibitor for MS in 1 M HCl solution. The change in corrosion potential (*E_corr_*) of a cathodic or anodic inhibitor must be greater than 85 mV from the uninhibited system. In contrast, this change is generally less than 85 mV in the case of a mixed inhibitor. The displacement measured in this research was lower than 85 mV, which indicates that they function as inhibitors of both types [16,23].

### 2.5. Effect of Temperature

**Figure 7:**
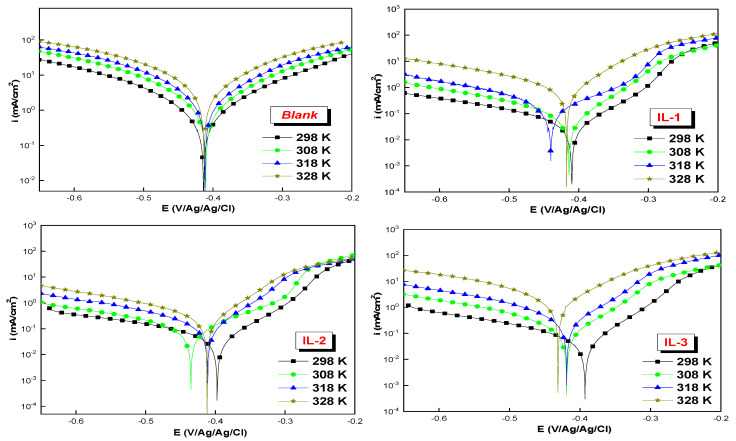
PDP plots for MS in 1 M HCl in the presence and absence of three ILs in 10^−3^ M at different temperatures.

The potentiodynamic polarization (PDP) method was employed to evaluate the effect of temperature on the corrosion behavior of mild steel (MS) in a 1 M HCl solution, in the absence and presence of 10^−3^ M of inhibitor, across a temperature range of 298 K to 328 K. Table 5 and Figure 7 present the electrochemical parameters and PDP plots of the mild steel (MS) in the presence of the ionic liquid inhibitors. Based on the results, we conclude that increasing temperature leads to a decrease in E_PDP_, while i_corr_ increases with increasing temperature due to the acceleration of the electrochemical reactions and the formation of stronger chemical bonds via adsorption [20]. As the temperature rises, the inhibitor adsorption equilibrium shifts to the desorption process, resulting in decreased surface coverage and a decrease in protection against corrosion diffusion on the surface.

The evaluation of multiple thermodynamic activation parameters was based on the Arrhenius equations and their modified versions. Specifically, the activation energy (*E_a_*), entropy (Δ*S_a_*), and enthalpy (Δ*H_a_*) are measured during the steel corrosion of the mild steel in a 1 M solution of HCl, with and without the synthesized inhibitor. An improved comprehension of the corrosion inhibition mechanisms is made possible by these thermodynamic data. This is the traditional Arrhenius equation and its reformulation based on the theory of the transition state [31,32].

$$i_{corr}=Aexp(-\frac{E_{a}}{RT})$$

$$\frac{E_{corr}}{T}=\frac{R}{Nh}\exp\left(\frac{\Delta S_{a}}{R}\right)exp(\frac{\Delta H_{a}}{RT})$$

**Figure 8:**
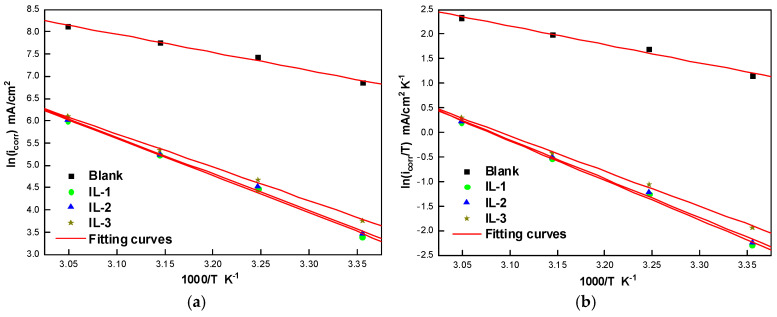
Arrhenius plots: (**a**) ln i_corr_ versus 1000/T, (**b**) ln *i_corr_*/T versus 1000/T for MS in 1 M HCl with and without synthesized ILs.

Figure 8 shows the Arrhenius curves showing ln i^0^ (corrosion) and ln(i^0^/T) as a function of 1000/T. From the slopes and orthogonals that are at the origin of these curves, the activation energy (*E_a_*), enthalpy (Δ*H_a_*), and entropy (Δ*S_a_*) were determined and are shown in Table 6. The results of this research show that the values of *E_a_* obtained with the existence of the tested inhibitors exceed those measured in a 1 M HCl solution without an inhibitor. It can be argued that this increase is caused by the physical adsorption of the inhibitors on the metal mild steel surface.

In addition, the high and positive values of Δ*H_a_* show the endothermic character of the steel dissolution mechanism (MS). Also, the lower negative values of ΔS_a_ observed in the presence of IL-3 and the positive values of IL-2 and IL-1, compared with the acid solution without an inhibitor, suggest an increase in the molecular order when the reagents are adsorbed to the steel/solution interface.

### 2.6. Surface Characterization (SEM-EDS)

**Figure 9:**
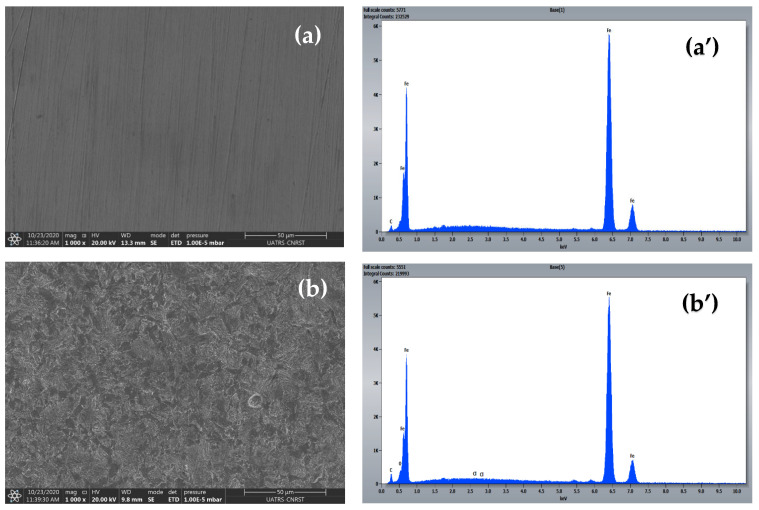

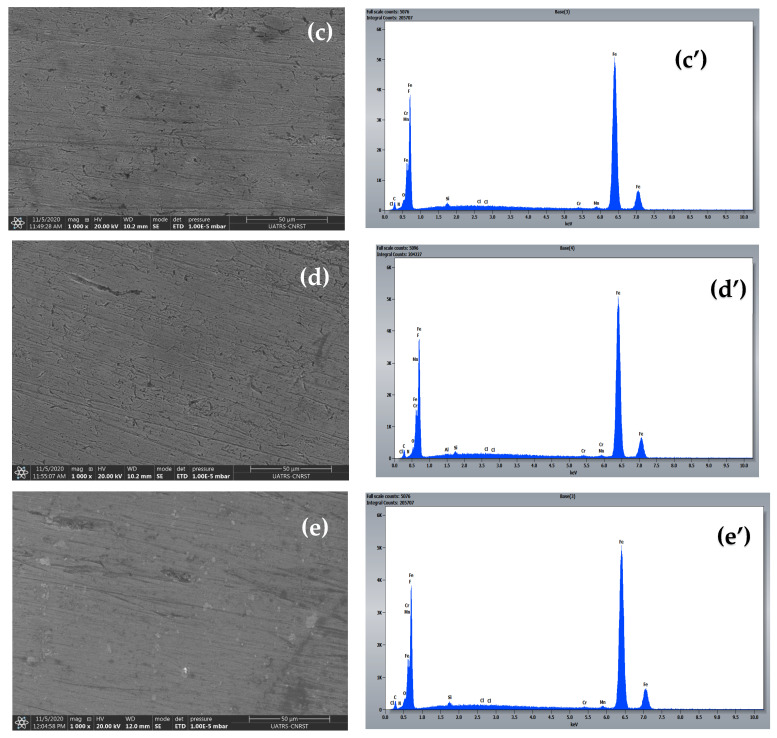
Surface morphology and corresponding EDX analysis of MS samples: (**a**,**a’**) Polished, (**b**,**b’**) Immersed in 1 M HCl (blank), and treated with (**c**,**c’**) IL-1, (**d**,**d’**) IL-2, and (**e**,**e’**) IL-3.

The SEM images of the metal mild steel specimens are shown in Figure 9. Figure 9a represents the surface of the polished MS, a non-corroded surface with some scratches due to the polishing. However, after 6 h of immersion in 1 M HCl, the surface morphology was strongly corroded and damaged with cracks and pits (Figure 9b). On the contrary, the mild steel surface damage is considerably lower, and the surface of the metal is relatively smooth with the existence of the protection of inhibitors (Figure 9c–e).

The EDX spectra give a general idea about the surface composition of the metal. In this study, we use the EDX of MS specimens before and after immersion in 1 M HCl in the presence and absence of the studied inhibitors (Figure 9). The EDX spectra display peaks corresponding to the primary elements found in the MS (Fe and C). In Figure 9b’, after the immersion in HCl, we notice the appearance of the Cl resulting from the adsorption of HCl, while the presence of N and Cl with the addition of inhibitors is shown in Figure 9c’–e’. These provide evidence for the existence of the inhibitors on the mild steel surface [33,34].

### 2.7. Theoretical Information

**Figure 10:**
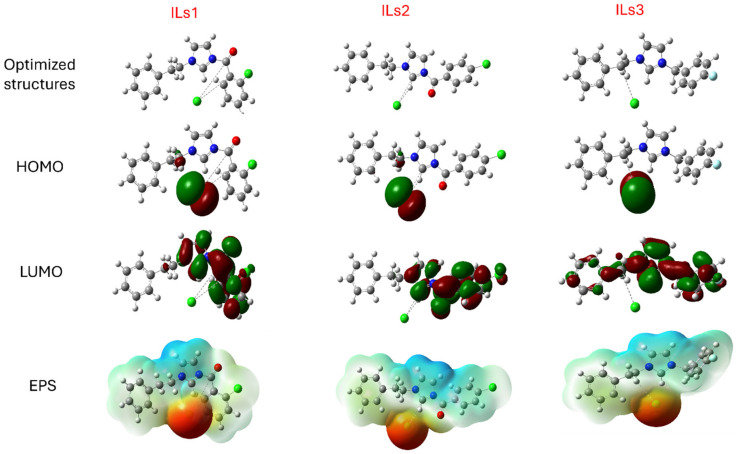
Optimized structures, HOMO, LUMO, and EPS of the studied ionic liquid inhibitors.

The ionic liquid optimization structures are presented in Figure 10. These geometry optimization structures are optimized using the B3LYP and 6-311 G (d,p) bases in the aqueous phases. The HOMO and LUMO orbitales and EPS of the ionic liquids are shown in Figure 10. The HOMO density distributed over the structure regions of the ionic liquids is responsible for the electron’s donation capacity. Also, these regions of LUMO density are the center responsible for electron acceptance from the surface of the substrate [35,36]. The parameters calculated for the synthesized ionic liquids, HOMO and LUMO energy, and gap energy, are described in Table 7.

The high E_HOMO_ value refers to the ability to donate free electrons to a vacant orbital of the metals. And the lower E_LUMO_ value indicates the strong tendency of the molecules to gain electrons. The three synthesized ionic liquids have the same E_HOMO_, with a small difference, giving them the following ranking: ILS2 > ILS1 > ILS3. This reflects that ILS2 can share its electrons more than the other ionic liquids. Additionally, the gap energy parameter is crucial for assessing the inhibitory activity of the molecules. A smaller gap energy indicates better reactivity of the inhibitors against the corrosion rate. In our study, ILs 1 and ILs 2 have the lowest gap energy values, 0.1402 and 0.1437, respectively, which can be explained by the superior adsorption potential of ILs 1 and 2 on the surface of the mild steel. Also, the fraction of transferred electrons, ΔN, is an essential indicator of the corrosion inhibition efficiencies of the molecules. ΔN > 0 describes a great donation capacity of electrons, which can decrease the effect of corrosion on the surface of metals [36,37].

The ΔE_b–d_ (backdonation energy) also provides insight into the stability of our molecule; a negative value of ΔE_b–d_ indicates that the structure is more stable. In our case, the calculated values for ΔE_b–d_ are −0.0175 eV for Ils1, −0.0179 eV for Ils2, and −0.0256 eV for Ils3, which are caused by the stabilization of these ionic liquid molecules. The electronegativity parameter is linked to the capability to donate electrons. The highest electronegativity value of an inhibitor corresponds to its lowest capacity to provide electrons. The electronegativity parameter is linked to the capability to donate electrons. The highest electronegativity value of an inhibitor corresponds to its lowest capacity to provide electrons. In the present study, the electronegativity values of our inhibitors are −0.1642, −0.1631, and −0.1312 for IL1, ILs 2, and ILs 3, respectively. These align with our results. The DFT calculation parameters display harmony with the experimental results.

**Figure 11:**
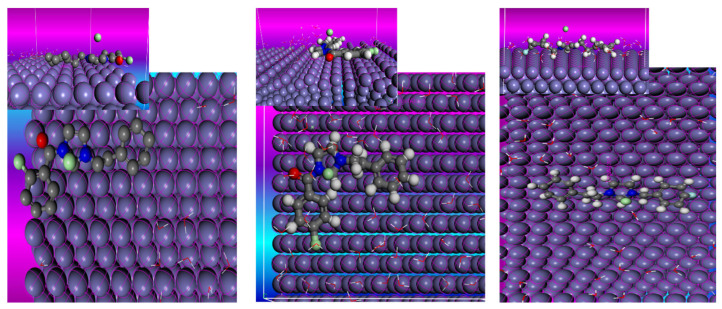
The top and side views of the most stable energy configuration of the Il inhibitors on the Fe(1 1 0) surface.

To study the interaction between the synthesized ionic liquids and the mild steel surface, a Monte Carlo simulation was employed as an effective computational method. Figure 11 shows the most stable adsorption configurations for the synthesized inhibitors based on the ionic liquids on the surface of the Fe(1 1 0) in the water solution. It is clear from the top and side views of the IL molecules on the surface of the Fe(1 1 0) that the molecules are oriented parallel to the iron surface, which represents the best position for adsorption. The highly negative adsorption energy values represent the higher efficiency of inhibition, which is due to the stable and strong chemical bond formed between the inhibitors and the surface of the Fe(1 1 0). The values of the adsorption energy for the ionic liquid molecules are given in Table 8. We can explain the mechanism of adsorption through the nitrogen, oxygen, and pi electrons of the aromatic ring; these donating electrons exist in inhibitors, which can occupy the empty d orbitals, creating a protective film on the mild steel surface. The adsorption energies of our inhibitors have high negative values, −3145.745 for ILS1, −2980.188 for ILS2, and −2099.160 for ILs3, thus showing that the strong inhibition efficiency of our inhibitors is in good agreement with the experimental results obtained from the PDP and EIS techniques.

## 3. Materials and Methods

### 3.1. Synthesis of Imidazolium-Derived (IL-1, IL-2, and IL-3)

**Figure 12:**
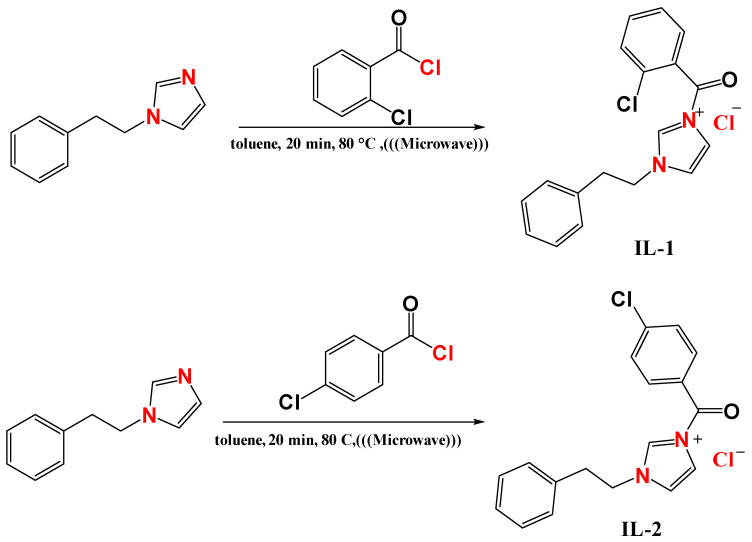

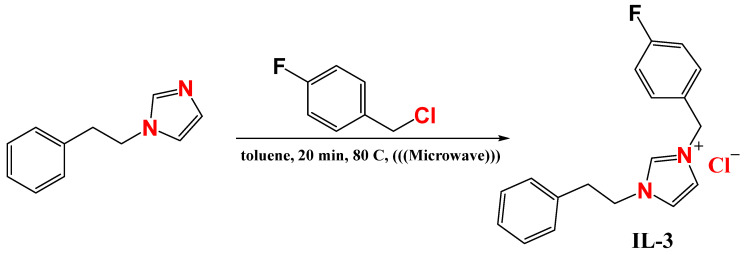
Procedure to synthesize imidazolium chloride inhibitors (IL-1, IL-2, IL-3).

The synthetic route for the preparation of the imidazolium-based ionic liquid derivatives is illustrated in Figure 12. In a typical procedure, the alkyl halides—2-chlorobenzoyl chloride, 4-chlorobenzoyl chloride, and/or 1-(chloromethyl)-4-fluorobenzene (1.1 eq)—were added to a solution of 1-phenethyl-1H-imidazole (1.0 eq) in toluene. The reaction mixture was then irradiated at 80 °C for 20 min in a sealed vessel using a SEM microwave reactor (CEM Corp., Matthews, NC, USA). Upon mixing, a clear and homogeneous solution was observed, indicating the formation of an oily intermediate. The resulting product was extracted using ethyl acetate. All the synthesized derivatives were then dried under reduced pressure.

### 3.2. Mild Steel and Solutions Preparation

Samples of mild steel (0.21% C, 0.38% Si, >1% of Mn, S, P, Al, and balance by Fe) were analyzed using electrochemical analysis. For all the studies, an aggressive 1 M hydrochloric acid solution was used. All these samples were mechanically polished using various emery papers and then rinsed with methanol and water, before drying. The electrolyte solution was prepared using 37% hydrochloric acid (HCl). The concentration of the studied imidazolium IL ranged from 10^−5^ to 10^−3^ M. This work used no supporting solvent to dissolve the corrosion inhibitors directly in a 1 M HCl solution.

### 3.3. Characterization of IL-1, IL-2 and IL-3

#### 3.3.1. The 3-(2-Chlorobenzoyl)-1-phenethyl-1H-imidazol-3-ium chloride (IL-1)

The 3-(2-chlorobenzoyl)-1-phenethyl-1H-imidazol-3-ium chloride (IL-1) can be characterized as follows. FT-IR, cm^−1^: *ν* = 765 (C-H, CH_2_), 1154 (C-N), 1561 (C=N), 1672 (C=O), and 2674 and 3100 (Ar-H). ^1^H NMR (400 MHz, CDCl_3_) (Figure 12): δH = 3.16 (t, 2H, CH_2_), 4.55 (t, 2H, CH_2_), 7.14–7.74 (d, 2H, Ar-H), 7.01–7.40 (m, 9H, Ar-H), and 9.27 (s, 1H, Ar-H); ^13^C NMR (100 MHz, CDCl_3_) (Figure 12): δC = 36.7 (CH_2_), 50.9 (CH_2_), 119.6 (CH), 121.1 (CH), 127.5 (CH), 128.3 (CH), 128.6 (CH), 129.3 (CH), 131.3 (CH), 131.8 (CH), 134.9 (CH), 135.7 (C), 137.1 (C), 137.3 (C), 137.7 (CH), and 173.3 (CO); Found: C, 62.32, H, 4.57, N, 8.14%. Calcd. for C_18_H_16_Cl_2_N_2_O, C, 62.26, H, 4.64, N, 8.07%.

#### 3.3.2. The 3-(4-Chlorobenzoyl)-1-phenethyl-1H-imidazol-3-ium chloride (IL-2)

**Figure 13:**
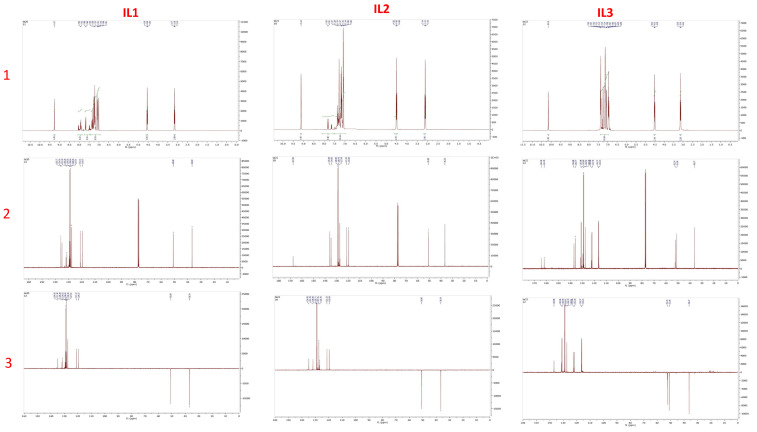
^1^H NMR spectrum (**1**), ^13^C NMR spectrum (**2**) in CDCl_3_ (400 MHz), and DEPT-135 NMR spectrum in CDCl_3_ (100 MHz) (**3**) of IL-1, IL-2, and IL-3.

The 3-(4-chlorobenzoyl)-1-phenethyl-1H-imidazol-3-ium chloride (IL-2) can be characterized as follows. FT-IR, cm^−1^: *ν* = 747 (C-H, CH_2_), 1154 (C-N), 1561 (C=N), 1707 (C=O), and 2687 and 3100 (Ar-H). ^1^H NMR (400 MHz, CDCl_3_) (Figure 13): δH = 3.12 (t, 2H, CH_2_), 4.50 (t, 2H, CH_2_), 7.22–7.85 (d, 2H, Ar-H), 7.01–7.39 (m, 9H, Ar-H), and 9.27 (s, 1H, Ar-H); ^13^C NMR (100 MHz, CDCl_3_) (Figure 12): δC = 36.5 (CH_2_), 50.8 (CH_2_), 119.6 (CH), 121.3 (CH), 126.7 (CH), 127.3 (CH), 128.9 (CH), 131.8 (CH), 134.9 (CH), 135.7 (C), 137.1 (C), 137.3 (C), 137.7 (CH), and 167.6 (CO); Found: C, 62.35, H, 4.58, N, 8.12%. Calcd. for C_18_H_16_Cl_2_N_2_O, C, 62.26, H, 4.64, N, 8.07%.

#### 3.3.3. The 3-(4-Fluorobenzyl)-1-phenethyl-1H-imidazol-3-ium chloride (IL-3)

The 3-(4-fluorobenzyl)-1-phenethyl-1H-imidazol-3-ium chloride (IL-3) can be characterized as follows. FT-IR, cm^−1^: *ν* = 751 (C-H, CH2), 1150 (C-N), 1508 (C=N), and 2843 and 3135 (Ar-H). ^1^H NMR (400 MHz, CDCl_3_) (Figure 13): δH = 3.14 (t, 2H, CH2), 4.51 (t, 2H, CH2), 5.46 (s, H, CH2), 7.21–7.53 (d, 2H, Ar-H), 6.90–7.41 (m, 9H, Ar-H), and 10.15 (s, 1H, Ar-H); ^13^C NMR (100 MHz, CDCl_3_) (Figure 12): δC = 36.3 (CH2), 51.0 (CH2), 52.2 (CH2), 116.1 (CH), 116.4 (CH), 121.7 (CH), 122.4 (CH), 128.7 (CH), 128.9 (CH), 130.9 (CH), 131.2 (C), 135.6 (C), 136.9 (CH), and 167.6 (C); Found: C, 68.32, H, 5.68, N, 8.90%. Calcd. for C_18_H_18_ClFN_2_, C, 68.24, H, 5.73, N, 8.84%.

### 3.4. Electrochemical Measurements

To carry out the electrochemical tests, we need a mild steel used as a working electrode with a surface area of 1 cm^2^, a counter electrode consisting of platinum (Pt), and also a reference electrode consisting of Ag/AgCl. The EIS measurement was performed with an amplitude of 5 mV, a frequency range of 100 kHz to 100 MHz, using an AC signal at the open-circuit potential (OCP). All the experiments were conducted at ambient temperature, without agitation. The curves of polarization were established according to the potential, ranging between −250 and +250 mV, with respect to the OCP. After reaching a scan speed of 1 mV/s, the OCP was stabilized for 30 min. The equations below can estimate the inhibition efficiency of each inhibitor [38,39].

$${EI}_{EIS}=\frac{R_{C}-R_{C}^{0}}{R_{C}}$$

$${EI}_{PDP}=\frac{i_{corr}^{0}-i_{corr}}{i_{corr}^{0}}$$

### 3.5. Surface Characterization

The mild steel specimens underwent a 6 h immersion in 1 M HCl at room temperature and 10^−3^ M of inhibitors for surface analysis. The Quattro ESEM-FEG is a type of scanning electron microscope (SEM) (Thermo Fisher Scientific, Waltham, MA, USA) that combines Environmental Scanning Electron Microscopy (ESEM) and Field Emission Gun (FEG) technologies. This technique was used to obtain SEM images by assessing the accelerating voltage of 20 kV at 1000× magnification, associated with X-ray photoelectron spectroscopy (EDX) (Thermo Fisher Scientific, Waltham, MA, USA).

### 3.6. Theoretical Details

#### 3.6.1. Density Functional Theory (DFT)

The DFT methods were calculated using Gaussian 09 and Gauss view software 6.0 [40], with a hybrid function of the Beeke parameters Lee, Yang, and Parr B3LYP and G-311G (d,p) comes basis set [41] was also calculated in water utilizing the integral equation formalism variant of the polarizable continuum module (IEFPCM). The properties of the molecules via the energies of the highest occupied molecular orbital E_HOMO_ and lowest unoccupied molecular orbital *E_LUMO_* can be explained using the quantum chemical descriptor premised on the default calculation. These descriptors can be calculated using the HOMO and LUMO energies, including the energy gap (Δ*E_gap_*), harness (*η*), softness (*σ*), chemical potential (*µ*), electronegativity (*χ*), electrophilicity (*ω*), nucleophilicity (*ε*), fraction of electrons transferred (Δ*N*), and back donation energy (Δ*E_b–d_*), and they were calculated as follows [42,43,44]:

$$\Delta Egap=E_{LUMO}-E_{HOMO}$$

$$\eta =\frac{\Delta Egap}{2}=\frac{E_{LUMO}-E_{HOMO}}{2}$$

$$\sigma =\frac{1}{\eta }=\frac{2}{E_{LUMO}-E_{HOMO}}$$

$$\chi =\frac{E_{LUMO}+E_{HOMO}}{2}$$

$$\omega =\frac{\chi^{2}}{2\eta }$$

$$\Delta N=\frac{\Phi_{Fe}-\chi_{inh}}{2(\eta_{Fe}+\eta_{inh})}$$

$$\varepsilon =\frac{1}{\omega }$$

$${\Delta E}_{b–d}=-\frac{\eta }{4}$$

The *Φ* value for the Fe (1 1 0) surface is 4.82 eV and $\eta_{Fe}$ is null.

#### 3.6.2. Monte Carlo Simulation (MC)

To study the interaction of the ILs with the surface of the mild steel, we used the MC simulation in the Biovia Material Studio 2020 software. The geometry optimization of the surface of the Fe was explored using GGA/PBE on the castep module, and the ILs were explored using the Dmol3 (GGA, PBE). We used the Fe (1 1 0) surface due to its higher stabilization energy and packed surface [45]. The adsorption locator module was exploited to investigate how these molecules interacted and reacted with the surface of the metal, we used the compass force field [46,47] for the adsorption study because of its effectiveness in the field of corrosion modeling [15]. The crystal of the Fe was constructed with an edge of 60 A to ascertain that enough depth was achieved, and the 10 × 10 super cell. The adsorption study was carried out under solvation conditions using ILs + 100 H_2_O + 3 H_3_O^+^ + 3Cl^−^. The optimization structures of the ILs, Fe, H_2_O, H_3_O^+^, and Cl^−^ were calculated to obtain the most stable adsorbed configuration.

## 4. Conclusions

The effect of the ionic liquids IL-1, IL-2, and IL-3 on the inhibition of mild steel corrosion in 1 M HCl solutions was investigated. The results showed that the ionic liquids act as effective corrosion inhibitors. Additionally, the adsorption of the compounds IL-1, IL-2, and IL-3 in 1 M HCl solution followed the Langmuir isotherm, and they also function as mixed-type corrosion inhibitors. Furthermore, the theoretical calculations (DFT) and MC simulations produced results consistent with the experimental findings. The results indicated that IL-1 and IL-2 exhibited the highest inhibition efficiencies, with 96.6% and 96.9%, respectively.

## Figures and Tables

**Table 1:** EIS parameters for MS in 1 M hydrochloric acid in the absence and presence of studied ILs in various concentrations.

Medium	Conc. (M)	R_s_ (Ω cm^2^)	R_ct_ (Ω cm^2^)	CPE		Q (µF S^n−1^)	Ɵ	*ƞ*_imp_ %
				C_dl_ (µFcm^−2^)	n_dl_			
Blank	---	1.7	33	89.1	0.784	312.70	---	---
IL-1	10^−5^	1.8	116.7	44.2	0.830	107.7	0.717	71.7
	5 × 10^−5^	1.8	216.4	33.2	0.825	78.7	0.847	84.7
	10^−4^	1.9	404.5	18.4	0.793	50.5	0.918	91.8
	5 × 10^−4^	1.3	700.9	15.7	0.769	47.4	0.953	95.3
	10^−3^	1..0	989.0	12.2	0.703	45.3	0.966	96.6
IL-2	10^−5^	1.9	165.8	26.2	0.827	66.8	0.801	80.1
	5 × 10^−5^	1.7	210.6	24.5	0.815	64.7	0.843	84.3
	10^−4^	1.5	384	19.6	0.825	46.2	0.914	91.4
	5 × 10^−4^	1.1	790	11.9	0.756	37.3	0.958	95.8
	10^−3^	0.8	1081	8.8	0.702	35.2	0.969	96.9
IL-3	10^−5^	1.6	95.3	52.8	0.791	159.0	0.654	65.4
	5 × 10^−5^	1.8	107.8	43.3	0.828	108.4	0.694	69.4
	10^−4^	1.6	202.3	30.5	0.824	74.5	0.837	83.7
	5 × 10^−4^	1.8	402.4	18.5	0.818	45.1	0.918	91.8
	10^−3^	1.3	616.5	17.0	0.769	43.2	0.946	94.6

**Table 2:** EIS thermodynamic parameters obtained by using the Langmuir isotherm at 298 K.

Compound.	K (L/mol)	ΔG_ads_ (KJ/mol)	R^2^	Slopes
IL-1	16.13 × 10^4^	−39.6	0.99999	1.03
IL-2	16.80 × 10^4^	−39.7	0.99998	1.02
IL-3	7.03 × 10^4^	−37.6	0.99988	1.04

**Table 3:** EIS parameters for MS in 1 M hydrochloric acid in the absence and presence of studied ILs after various immersion times.

Medium	Time (h)	R_s_ (Ω cm^2^)	R_ct_ (Ω cm^2^)	CPE		Q (µF S^n−1^)	*η*_EIS_ %
				C_dl_ (µF cm^−2^)	n_dl_		
Blank	½	1.7	33.0	89.1	0.784	312.70	--
	1	1.6	26.4	122.7	0.810	364.90	--
	2	1.6	26.3	122.8	0.810	364.80	--
	4	1.5	21.4	267.0	0.834	627.00	--
	6	1.0	19.4	349.4	0.796	963.80	--
	12	1.2	10.4	419.5	0.764	949.2	--
IL-1	½	1.0	989	12.2	0.703	45.3	96.6
	1	1.2	943	15.4	0.737	48.9	97.2
	2	1.3	909	18.3	0.739	59.0	97.2
	4	1.8	784	22.6	0.767	66.8	97.3
	6	1.6	715	25.0	0.800	73.2	97.3
	12	1.3	615	30.8	0.826	80.9	98.3
IL-2	½	0.8	1081	8.8	0.702	35.2	96.9
	1	0.5	1280	7.5	0.747	32.6	97.9
	2	0.8	998	11.8	0.737	37.8	97.4
	4	1.0	853	13.0	0.734	43.1	97.4
	6	0.7	754	14.9	0.724	51.6	97.4
	12	1.2	635	18.2	0.722	62.9	98.3
IL-3	½	1.3	616.5	17.0	0.769	43.2	94.6
	1	1.6	555.2	20.2	0.791	57.6	95.2
	2	1.6	457.5	21.7	0.792	56.5	94.2
	4	1.4	386.4	26.0	0.798	65.9	94.4
	6	1.5	258.0	28.8	0.799	76.9	92.5
	12	1.2	179.8	34.6	0.786	102.6	94.2

**Table 4:** EIS parameters obtained by the concentration effect.

Medium	Conc. M	−E_corr_ mV/Ag/AgCl	i_corr_ µA cm^−2^	−β_c_ mV dec^−1^	β_a_ mV dec^−1^	*η*_PDP_ %
1 M HCl	---	413	944	139	128	----
IL-1	1 × 10^−5^	412	263	124	108	72.1
	5 × 10^−5^	410	137	127	112	85.5
	1 × 10^−4^	411	77	129	114	91.8
	5 × 10^−4^	409	38	133	122	95.9
	1 × 10^−3^	410	30	135	125	96.8
IL-2	1 × 10^−5^	419	172	127	112	81.8
	5 × 10^−5^	420	135	128	113	85.7
	1 × 10^−4^	413	83	132	122	91.2
	5 × 10^−4^	398	36	135	125	96.2
	1 × 10^−3^	397	32	138	126	96.6
IL-3	1 × 10^−5^	415	335	130	121	64.5
	5 × 10^−5^	417	282	134	118	70.1
	1 × 10^−4^	411	152	136	119	83.8
	5 × 10^−4^	410	83	135	116	91.2
	1 × 10^−3^	393	43	132	120	95.4

**Table 5:** Electrochemical parameters obtained under 4 temperatures with and without ILs.

Medium	Temperature K	−E_corr_ mV/ECS	i_corr_ µA cm^−2^	−β_c_ mV dec^−1^	β_a_ mV dec^−1^	*η*_PDP_ %
Blank	298	413	944	139	128	---
	308	410	1690	137	129	---
	318	411	2328	126	125	---
	328	412	3387	120	133	---
IL-1	298	410	30	135	125	96.8
	308	414	88	127	127	94.7
	318	440	187	135	119	91.9
	328	416	402	122	128	88.1
IL-2	298	397	32	138	126	96.6
	308	436	92	135	124	94.5
	318	411	190	130	128	91.8
	328	412	413	132	123	87.8
IL-3	298	393	43	132	120	95.4
	308	419	107	130	123	93.7
	318	418	208	131	122	91.0
	328	431	442	127	126	86.9

**Table 6:** Values of *E_a_*, Δ*H_a_*, and Δ*S_a_* without and with treatment of IL compounds.

Medium	E_a_ (KJ/mol)	ΔH_a_ (KJ/mol)	ΔS_a_ (J/mol K)
Blank	33.8	31.2	−82.7
IL-1	69.5	66.9	8.5
IL-2	68.5	65.7	5.0
IL-3	62.2	59.6	−13.2

**Table 7:** DFT calculated chemical parameters of the Ils molecules.

Parameters	Ils 1	Ils 2	Ils3
E_HOMO_	−0.2343	−0.2350	−0.2336
E_LUMO_	−0.0941	−0.0913	−0.0288
ΔE_gap_ (eV)	0.1402	0.1437	0.2048
η (eV)	0.0701	0.0718	0.1024
σ (eV^−1^)	14.2653	13.9275	9.7656
χ (eV)	−0.1642	−0.1631	−0.1312
ω	0.1923	0.1852	0.0840
ε	5.2000	5.3995	11.9047
ΔN_110_	35.5506	34.7012	24.1757
ΔE_b–d_ (eV)	−0.0175	−0.0179	−0.0256

**Table 8:** Monte Carlo simulation (MC) of Il inhibitors on Fe(110) surface.

Structures	Total Energy	Adsorption Energy	Rigid Adsorption Energy
Fe(1 1 0)@ILs 1@H_2_O	−2459.130	−3145.745	−2521.515
Fe(1 1 0)@ILs 2@H_2_O	−2293.572	−2980.188	−2347.356
Fe(1 1 0)@ILs 3@H_2_O	−2029.694	−2099.160	−2088.581

## Data Availability

Data will be made available on request.
